# Taste priming shapes online moral judgment: implications for cyberspace governance

**DOI:** 10.3389/fpsyg.2025.1495798

**Published:** 2025-03-26

**Authors:** Xianchao Huang, Shiying Zang, Jingxuan Wang, Yifan Zheng, Zhuolan Bai, Jinfeng Huang

**Affiliations:** ^1^School of Law, Tianjin University, Tianjin, China; ^2^Department of Psychology, Hebei Normal University, Shijiazhuang, China; ^3^College of Management and Economics, Tianjin University, Tianjin, China; ^4^Nanlian School, Longgang, Shenzhen, China

**Keywords:** taste perception, online moral judgments, tastelessness effect, bitterness effects, cyberspace governance

## Abstract

This study explores the link between taste perception and moral judgment, focusing on how tastelessness and varying taste intensities influence the assessment of online events. Participants were exposed to taste priming, ranging from tastelessness to mild and intense sweetness, as well as mild and intense bitterness, to evaluate their moral judgments on events with varying degrees of morality. The findings revealed no significant difference between the tasteless and sweet priming groups. However, the bitterness group exhibited complex effects: moderate bitterness led to the harshest judgments of obvious immoral events, while intense bitterness resulted in stricter judgments for moral events and more lenient judgments for immoral ones. These results suggest that tastelessness may mimic the effects of sweetness, and the influence of bitterness varies with its intensity. The study offers a new perspective on cyberspace governance, suggesting that regulating taste-related stimuli could influence online moral judgment and decision-making processes.

## 1 Introduction

Online platforms frequently expose us to incidents of cyberbullying, such as during international events like the Olympic Games, where athletes face harassment from various online communities. This raises key questions: what factors drive individuals to post morally questionable or legally challenging comments online? Under what circumstances are these behaviors more likely to occur, and how should they be addressed and regulated?

With the rapid development of internet technology and the widespread adoption of online platforms, cyberspace has become an indispensable part of people’s daily lives. Due to the varying levels of literacy among netizens and the inadequacy of legal regulations (van Eeten and Mueller, 2013), behaviors that violate online ethics or even challenge the law are increasingly common. Citizens often make value judgments on online events based on their moral principles and past experiences, assessing them on a spectrum of “negative or positive” (Cannon et al., 2011) and “good or bad” (Zalla et al., 2011)—a process known as moral judgment (Malle, 2021). These judgments, in turn, influence their behavioral tendencies and attitudes. In online contexts, moral judgments are not solely based on rational thinking but are also influenced by emotional and sensory intuitions, such as mood and comfort (Haidt, 2001). Incorrect judgments can directly affect the nature of online speech and may even lead to cyberbullying. Therefore, when tracing the origins of online behaviors, it is essential not only to fully consider the objective environment and individual characteristics but also to give due attention to physiological factors.

Embodied cognition (Varela et al., 2017; Barsalou, 1999), a foundation for phenomenology and conceptual metaphor theory, offers a novel perspective on moral judgment. Embodied cognition theory asserts that the body is fundamental to cognitive processes, shaping perception, with the environment and experience being crucial for cognitive development (Hewett and Thonus, 2019). It emphasizes that, in addition to visual observation and information processing, we also understand the world through bodily sensations such as touch and taste. From the perspective of embodied cognition theory, moral thinking is also influenced by physiological sensations and environmental information. Moral cognition changes with sensory and environmental variations, and at the same time, moral cognition can also influence an individual’s physiological sensations and environmental cognition in return. Among these, taste, as a physiological sensation that often attracts attention, is not only an important basis for judging food quality, but also provides us with crucial sensory information to respond to environmental stimuli.

Taste perception not only shapes basic sensory experiences but also profoundly influences cognitive processes, judgment, and behavioral patterns (Spence, 2020; Cai et al., 2017; Sagioglou and Greitemeyer, 2016). At the genetic level, humans exhibit innate preferences for sweetness and aversion to bitterness (Lipsitt, 1979; Steiner, 1973), suggesting these gustatory responses are genetically encoded. On the psychological trait dimension, gustatory experiences demonstrate significant correlations with personality characteristics: short-term consumption of spicy foods elicits aggressive cognitions (Batra et al., 2017; Barsalou, 1999), while long-term preference for spiciness correlates with higher trait anger levels (Ji et al., 2013); conversely, sweet food intake enhances prosocial tendencies by reinforcing positive self-identity related to helpfulness (Hewett and Thonus, 2019). In decision-making domains, taste stimuli modulate risk preferences through neural mechanisms—acidic taste promotion of risk-taking behaviors (Spence, 2020) vs. bitter taste suppression of impulsive consumption (van Eeten and Mueller, 2013). Notably, taste cross-modal effects extend to consumer behavior: spicy diet not only elevates aggression but also alters subsequent choice diversity via sensory memory mechanisms (Cai et al., 2017).

The relationship between taste perception and moral judgment has been systematically investigated across behavioral and neurophysiological levels, revealing profound and complex interactions between these domains. Since the 18th century, David Hume posited that moral judgments originate from affective experiences, drawing a parallel to taste judgments—physiological disgust often signals moral disapproval. This theoretical framework aligns closely with Jonathan Haidt’s Social Intuitionist Model proposed in 2001 (Haidt, 2001), though empirical evidence was initially lacking. Modern neuroscience has provided robust evidence for this connection: in 2009, Chapman et al. (2009) Utilized electromyography scanning technology to demonstrate shared neural activation patterns between bitterness perception and moral disgust. Subsequent fMRI studies further confirmed that both stimuli activate overlapping brain regions, including the temporal and frontal cortices (Schaich Borg et al., 2008; Moll et al., 2005; Calder et al., 2001). Crucially, the dorsal anterior cingulate cortex has been identified as a regulatory hub, playing a pivotal role in moral judgment and economic decision-making (selfish vs. prosocial behaviors) (Schaefer et al., 2023). These findings not only deepen our understanding of the interplay between moral emotions and neural mechanisms but also suggest that moral cognition may originate from an evolutionarily conserved motivational system for avoiding hazardous foods.

There exists a bidirectional association between taste perception and moral judgment. Behavioral evidence demonstrates that bitter taste stimuli enhance punitive judgments toward immoral acts (Eskine et al., 2011), while genuine sweet taste experience promotes lenient evaluations and greater tolerance for retributive behavior (Hellmann et al., 2013); sweet taste has also been significantly correlated with prosocial behaviors (Schaefer et al., 2023; Schaefer et al., 2021). Olfactory perception similarly contributes to moral cognition: pleasant aromatics enhance prosocial tendencies, whereas aversive odors intensify moral condemnation (supplementary olfactory mechanisms). Such cross-modal interactions possess neurobiological substrates—moral conflicts strengthen gustatory disgust (Eskine et al., 2012), while unjust events activate primal defense systems and amplify sensory sensitivity (Skarlicki et al., 2013). Notably, moral content itself shapes gustatory experience: Perceptions of moral transgressions induce gustatory aversion, virtuous acts elicit pleasant sensations, and neutral events produce no significant effect (Eskine et al., 2012). Collectively, these findings establish a dynamic body-cognitive system where gustatory-moral bidirectionality continuously evolves through temporal interactions.

Given the significant impact of taste on moral judgment, the cross-modal association has important implications for the governance of cyberspace, particularly in addressing online behaviors like cyberbullying. Building on previous research, this study explores how these insights can be applied to cyberspace governance. Our study aims to address the following key questions:

First, the differential effects of sweet taste vs. tasteless conditions on moral judgment urgently require clarification. Eskine et al. (2011) indicated that there was no significant difference in moral judgment effects between sweetness and tastelessness, suggesting that sweetness effect might be replaceable by tastelessness. However, Hellmann et al. (2013) found that sweetness, compared to tastelessness, significantly increased tolerance toward revengeful behavior, challenging the former conclusion. Additionally, recent studies have used plain water as a baseline control but have not explored its specific role (Li et al., 2023). We use a between-subjects design to further investigate the differences among sweetness and tastelessness, proposing Hypothesis 1: There will be no significant difference in online moral judgments between participants who drink plain water and those who drink sweet beverages.

Second, the standardization of experimental designs demands immediate refinement to address methodological inconsistencies. Inconsistencies in the selection and description of priming materials have exacerbated the complexity of results. Early studies only briefly described the final taste without specifying its source (Hellmann et al., 2013), while another experiment used complex beverages such as Minute Maid Berry Punch and Swedish Bitters, making it difficult to rule out their non-specific effects and replicate the findings (Eskine et al., 2011). Although recent studies have shifted to using sucrose solutions and bitter coffee, potential interference from caffeine cannot be ignored (Li et al., 2023). The complex nature of these materials complicates the interpretation of results. We conducted a pilot experiment, selecting easily obtainable and single-ingredient materials, namely sucrose and bitter melon powder, to eliminate potential ingredient interference.

Moreover, the non-standardization of bitter and sweet agents’ ingredients leads to difficulties in comparing doses, and current research generally lacks strict dose control and standardized experimental procedures. The timing and quantity of beverage consumption have not been precisely controlled, affecting the reliability and reproducibility of the results (Eskine et al., 2011; Hellmann et al., 2013; Li et al., 2023). We hypothesize that this inconsistency partly stems from imprecise taste measurement and the lack of quantification standards, making it unclear how varying taste dosages specifically impact judgment results. Given the common choices of sugar-free, low-sugar, semi-sweet, standard sugar, and full/multiple sugars in everyday life, it is worth further exploring whether drinks with different sweetness levels lead to different results. We have established a standardized experimental procedure, strictly controlling the participants’ drinking timing, frequency, and dosage, and used preliminary experiments to identify three different concentrations for sweet and bitter tastes, all with simple and obtainable ratios. We propose Hypothesis 2: Different levels of taste intensity will differentially impact participants’ online moral judgments.

Third, the moderating role of Chinese cultural context in the gustatory-moral association awaits empirical validation. Taste perception is deeply influenced by cultural background. For example, whether findings on spiciness in specific cultural settings (such as India) are broadly applicable is debatable (Batra et al., 2017). The diversity of global food cultures and cultural differences in definitions of sweetness and bitterness limit the generalizability of research findings. Since our participants are from China, where taste has traditionally been associated with beauty and given unique aesthetic value, and where the philosophical focus is on the bodily experience of taste, we hold a unique interpretation of the culture of suffering, leading to Hypothesis 3: Different degrees of bitterness have differential effects on participants’ online moral judgments.

Fourth, the deficiency in ecological validity of current experimental paradigms necessitates remediation to bridge laboratory findings with real-world online behaviors. Moral events in online environments exhibit multifaceted complexity: they require not only differentiation between moral and immoral attributes but also consideration of event-specific typologies (e.g., defamatory remarks vs. information tampering) and severity gradients (e.g., mild offenses vs. severe discrimination). Current research predominantly relies on simplified paradigms (e.g., binary moral categorization tasks), which fail to capture the dynamic interplay between gustatory stimuli and moral judgment in real-world contexts. To address this limitation, our study integrates two critical dimensions into the experimental framework: event type (moral/immoral) and severity level (obvious/subtle), thereby enhancing ecological validity and contextual relevance. We hypothesize that taste may have different effects on online events of varying levels and types, proposing Hypothesis 4: Taste perception exerts a differential impact on the evaluation of different types of online events and varying levels of online event severity.

This study investigates the impact of gustatory stimuli (sweetness/bitterness) on online moral judgment through behavioral experiments, aiming to elucidate the interaction mechanisms between sensory experiences and cultural cognition. Addressing the limitations of existing research in delineating gustatory effect boundaries (sweetness/tastelessness controversies), standardizing experimental paradigms (material/dosage control), cultural universality (East–West differences in bitterness metaphors), and ecological validity (event type/severity dimensions), this study advances from three aspects: theoretical, methodological, and practical. Theoretically, this study, for the first time, assesses how the philosophical meanings of “bitterness” in Chinese culture impact moral judgments, thus questioning Western - centred assumptions. Methodologically, it devises a standardized experimental paradigm to boost its ecological validity. Practically, it puts forward a culturally adapted intervention framework, furnishing scientific evidence for cyberspace governance.

## 2 Materials and methods

### 2.1 Participants and design

The sample size for the study was calculated to be 91 participants using G*Power 3.1. In total, 105 students from Hebei Normal University were recruited to participate in the formal experimental study. Among them, 48 were male and 57 were female, with an average age of 20.10 years (SD = 1.21). Due to incomplete data, the final sample consisted of 98 participants, with an average age of 20.04 years (SD = 1.06) (45 males, M_age_ = 20.45, SD = 1.04; 53 females, M_age_ = 19.68, SD = 0.96). The research protocol followed the guidelines of the Declaration of Helsinki and was approved by the Biomedical Ethics Committee of Hebei Normal University. Written informed consent was obtained from each participant after an explanation of the nature and possible consequences of the study.

The experimental procedure was designed using E-prime 2.0. A 7 × 2 × 2 mixed-factorial design was adopted, with independent variables being taste type, event type, and event severity. The taste type had 7 levels: tastelessness, mild sweetness, moderate sweetness, intense sweetness, mild bitterness, moderate bitterness, and intense bitterness, which served as a between-subjects variable. Event type and event severity were within-subjects variables. Event type had 2 levels: moral and immoral, and each event type was further divided into two levels of event severity: slight and obvious. The dependent variable was the participants’ ratings of the events.

Since there were seven levels of the between-subjects factor (taste type), participants were randomly assigned to seven corresponding groups. The final groups distribution was as follows: tastelessness (plain water, 15 participants), mild sweetness (15 participants), moderate sweetness (14 participants), intense sweetness (13 participants), mild bitterness (14 participants), moderate bitterness (12 participants), and intense bitterness (15 participants). Efforts were made to ensure a roughly equal number of males and females in each group.

### 2.2 Materials

At the initial phase of the study, an additional 80 university students were recruited to participate in a pre-experiment to determine the experimental materials. Among them, 10 participants were involved in quantifying the taste-priming materials, while the remaining 70 participants were involved in selecting images for the online moral judgment task. For the taste-priming materials, volunteers participated in a taste-testing session to identify the three levels (mild, moderate, intense) of taste stimuli required for the experiment. The final taste-priming solutions were prepared by dissolving different amounts of sugar or bitter melon powder in 100 mL of plain water: 2.5 *g* of sugar for mild sweetness, 5 *g* for moderate sweetness, and 8.5 *g* for intense sweetness; 1.5 *g* of bitter melon powder for mild bitterness, 2.5 *g* for moderate bitterness, and 5.0 *g* for intense bitterness. The mixtures were well-stirred to ensure consistent taste stimuli.

For determining the materials for online moral judgment, a self-developed questionnaire was used. After collecting and analyzing the data, 70 questionnaires were administered using Wenjuanxing (a Chinese online survey platform), with 60 valid questionnaires selected. Based on the questionnaire results, irrelevant materials were eliminated, resulting in 80 images for the online moral judgment task, with 20 images corresponding to each level of stimulus (moral/immoral × mild/obvious).

Building on previous research (Li et al., 2023), we made improvements by incorporating a combination of text and images, with the images sourced from public image databases. The events reflected in these text-image pairs are commonly seen on the internet, thus enhancing the ecological validity of our approach.

### 2.3 Procedure

**Figure 1:**
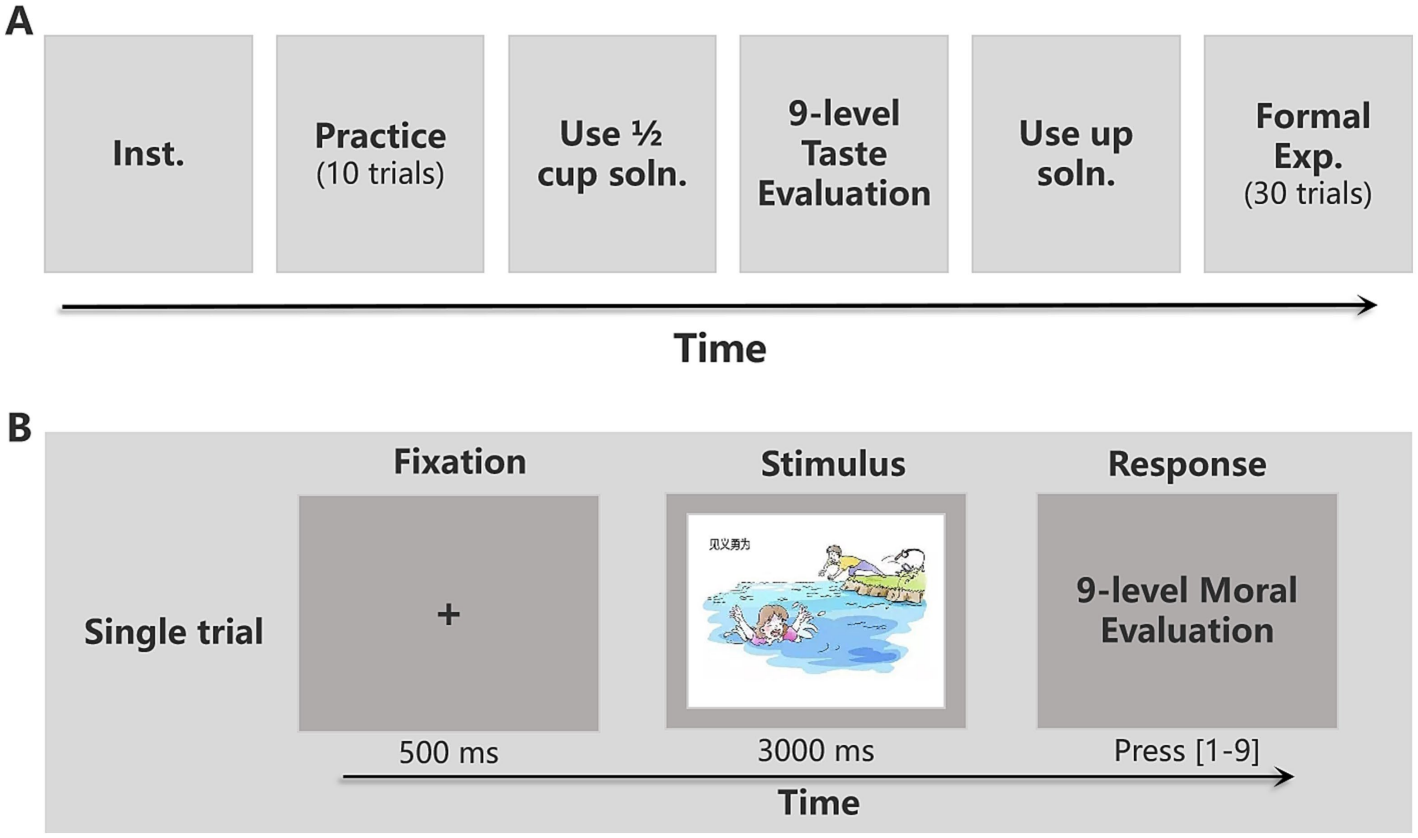
Overall experimental timeline and single-trial protocol. **(A)** Overall experimental timeline. **(B)** Single-trial protocol for the practice part and formal experiment part. The stimuli in the two parts are entirely different. The Chinese phrase in the stimulus picture is “见义勇为,” which means “Acting bravely for justice” (Scenario: a bystander rescues someone from drowning). Inst., Instructions; Soln., Solution; Exp., Experiment.

The experimenter explained the instructions to the participants and guided them through a practice phase consisting of 10 trials. The materials used in the practice phase were unrelated to the formal experiment. After the practice phase, participants entered the formal experiment. They were instructed to drink half of the taste-priming solution in the cup on the table and evaluate the taste using a nine-point rating scale, where 1 indicated no taste, and nine indicated intensely sweet or bitter, with the intensity of taste increasing from 1 to 9. After completing the taste evaluation, participants drank the remaining taste-priming solution and proceeded to make moral judgments on subsequent events, again using a nine-point scale where 1 represented very immoral, 5 represented neutral, and 9 represented very moral. There were 30 trials in total, with each image presented for 3 s, and participants had a time limit of 3 s to make their judgments. The overall experimental procedure and the time course of single trial are shown in Figure. 1.

## 3 Results

We conducted hierarchical analyses to disentangle the effects of taste priming across two critical dimensions: event type (moral vs. immoral) and severity level (mild vs. obvious).

### 3.1 Effects of taste priming on online moral judgment: event type

First, a one-way ANOVA was conducted with three major taste groups (tastelessness, sweetness, bitterness) as the independent variable to analyze the moral evaluation scores. The results were nearly significant [*F*_(2, 389)_ = 2.520, *p* = 0.082]. *Post-hoc* tests revealed a trend toward significant differences between the bitterness group and both the tastelessness (*p* = 0.052) and sweetness groups (*p* = 0.085). However, no significant difference was found between the tastelessness and sweetness groups (*p* = 0.499), which supports Hypothesis 1, indicating that there is no significant difference in online moral judgments between participants who drank plain water and those who drank sweet beverages.

**Figure 2:**
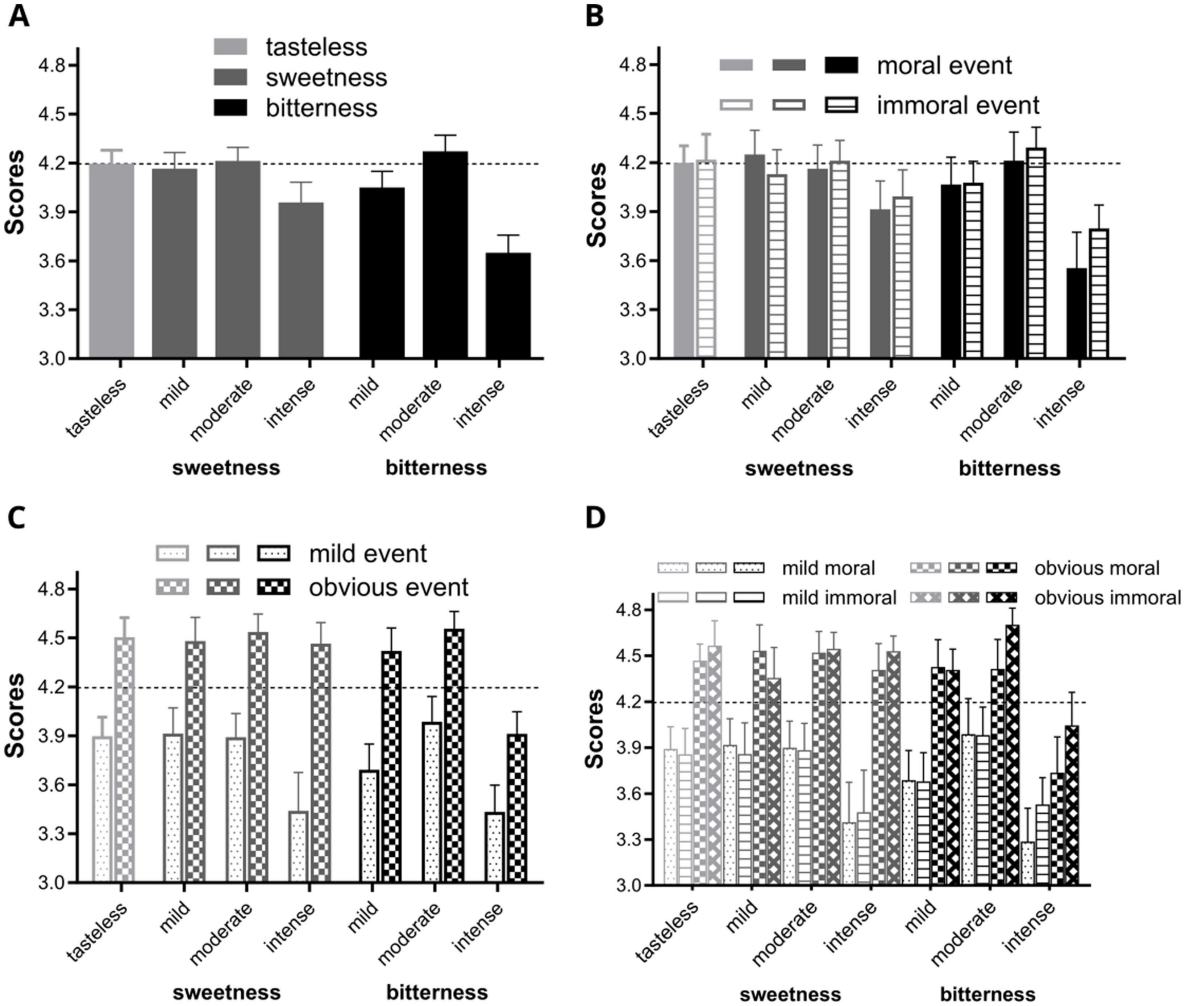
Rating results of online events under different taste priming conditions. In all panels, light gray denotes tastelessness condition, dark gray represents sweetness conditions, and solid black indicates bitterness conditions. **(A)** Ratings of online events across seven taste groups. **(B)** Ratings of online events categorized by event type under different taste priming (color-filled areas: moral events; horizontal line patterns: immoral events). **(C)** Ratings of online events categorized by event severity under different taste priming (small dot patterns: mild severity events; horizontal checkerboard patterns: obvious severity events). **(D)** Ratings of online events considering both event type and severity under different taste priming (small dot patterns: mild moral events; horizontal line patterns: mild immoral events; horizontal checkerboard patterns: obvious moral events; diagonal checkerboard patterns: obvious immoral events). The vertical axis of **A–D** represents the rating results, while the horizontal axis represents different taste types. The dashed line indicates the mean rating for participants with tastelessness priming (plain water, y = 4.19) in panel **(A)**. For clarity, the panels display ratings between 3.0 and 4.9, with error bars denoting standard errors.

Next, a one-way ANOVA was conducted with seven subgroup categories (tastelessness, mild sweetness, moderate sweetness, intense sweetness, mild bitterness, moderate bitterness, intense bitterness) as independent variables to analyze the evaluation scores of moral materials. The results showed significant differences in scores among the different taste types [*F*_(6, 385)_ = 4.658, *p* < 0.001], indicating that different taste types influence individuals’ moral judgments, as clearly illustrated in Figure 2A. As shown in Figure 2A, judgments of moral or immoral events were lowest under the intense bitterness condition (M = 3.65, SD = 0.85) and highest under the moderate bitterness condition (M = 4.27, SD = 0.69).

*Post-hoc* tests showed significant differences between the intense bitterness group and the other six groups: tastelessness, *p* < 0.001; mild sweetness, *p* < 0.001; moderate sweetness, *p* < 0.001; intense sweetness, *p* = 0.031; mild bitterness, *p* = 0.004; moderate bitterness, *p* < 0.001. Additionally, there was a significant difference between the intense sweetness and moderate bitterness groups, *p* = 0.038. These *post-hoc* test results demonstrate that different taste intensity stimuli can lead to different rating results, supporting Hypothesis 2. No differences were observed between the three sweetness groups and the plain water group, which further supports Hypothesis 1.

A repeated measures ANOVA was conducted on event type under different taste priming conditions, revealing no significant difference in scores between event types [*F*_(1, 91)_ = 0.699, *p* = 0.405]. Figure 2B shows the ratings of online events categorized by event type under different taste priming. As we can see from the panel, different taste primes have different effects in the moral and immoral groups. To further examine the effects of taste on different types of moral events, we separately analyzed the scores for moral and immoral events.

Analysis of scores for moral events. A one-way ANOVA revealed significant differences in participants’ ratings of moral events across different taste priming conditions [*F*_(6, 91)_ = 2.328, *p* = 0.039]. *Post-hoc* tests indicated significant differences between the intense bitterness group and the tastelessness group (*p* = 0.005), mild sweetness group (*p* = 0.003), moderate sweetness group (*p* = 0.010), mild bitterness group (*p* = 0.028), and moderate bitterness group (*p* = 0.007). As shown in Figure 2B, participants rated moral events most strictly under intense bitterness condition.

Analysis of scores for immoral events. In contrast, the one-way ANOVA results for immoral events did not show significant differences in ratings across different taste priming conditions [*F*(6, 91) = 1.369, *p* = 0.236]. However, *post-hoc* analysis revealed significant differences between the intense bitterness group and the tastelessness group (*p* = 0.035), moderate sweetness group (*p* = 0.041), and moderate bitterness group (*p* = 0.019). Figure 2B shows that under intense bitterness condition, participants were most tolerant of immoral events, while the most stringent ratings were given under the moderate bitterness condition.

Looking at both types of events together, we find that participants in the intense sweetness group gave lower scores for both moral and immoral events. In contrast, the intense bitterness condition exhibited greater leniency toward immoral events and stricter judgments toward moral events.

### 3.2 Effects of taste priming on online moral judgment: event severity

Next, we further analyzed the impact of event severity. Figure 2C shows the rating results after categorizing the event materials into two levels of severity: mild and obvious. As we can see, there are significant differences in the rating results between mild and obvious events. Therefore, we further explored the effect of event severity. With taste type as a between-subjects factor, we analyzed the effects of event type and event severity using a repeated measures ANOVA. The main effect of event severity was significant [*F*_(1, 91)_ = 147.668, *p* < 0.001], validating the event severity classification used in the study. No interaction effects were observed between variables. Figure 2D displays the rating results after responses were subdivided into four events types.

A one-way ANOVA was conducted to separately analyze the effects of taste types on participants’ scores under different event severity levels. For mildly severe events, the results showed a trend toward significant differences in participants’ scores across different taste priming conditions [*F*_(6, 91)_ = 1.984, *p* = 0.076]. *Post-hoc* analysis revealed significant differences between the intense sweetness group and the mild sweetness group (*p* = 0.044) and the moderate bitterness group (*p* = 0.029); significant differences were also found between the intense bitterness group and the tastelessness group (*p* = 0.041), mild sweetness group (*p* = 0.035), moderate sweetness group (*p* = 0.048), and moderate bitterness group (*p* = 0.022), thereby supporting Hypothesis 3, that different degrees of bitterness have differential effects on participants’ online moral judgments.

For obviously severe events, the results showed significant differences in participants’ scores under different taste priming conditions [*F*_(6, 91)_ = 3.170, *p* = 0.007]. *Post-hoc* analysis revealed significant differences between the intense bitterness group and all other groups: tastelessness (*p* = 0.001), mild sweetness (*p* = 0.002), moderate sweetness (*p* = 0.001), intense sweetness (*p* = 0.003), mild bitterness (*p* = 0.006), and moderate bitterness (*p* = 0.001). These results support Hypothesis 4, indicating that taste perception differentially impacts the evaluation on the evaluation of online events depending on their severity.

## 4 Discussion

This study demonstrates that taste priming significantly influences online moral judgments, with nuanced effects based on both taste type and intensity. Specifically, while tastelessness and sweetness were found to produce similar effects, bitterness showed a more complex impact on judgments. Moderate bitterness led to the harshest judgments of immoral events, whereas intense bitterness resulted in stricter assessments of moral events but more lenient judgments of immoral ones. Overall, taste priming did not produce differential effects on evaluations of moral vs. immoral events, but it did influence judgments based on the severity of the events.

### 4.1 Tastelessness = sweetness? New insights on the role of plain water

Our experimental findings reaffirmed the impact of taste perception on moral processing. Additionally, we explored an often-overlooked issue in previous studies—the role of tasteless beverages, particularly plain water. Drawing on previous studies, we identified instances where the effects of tasteless and sweet taste primes on subsequent judgments were indistinguishable, prompting the question: can certain sweetness-induced effects be replicated by tastelessness? Our study supported this hypothesis, as we found no significant difference between the tastelessness group and the sweetness group, even when examining seven distinct taste categories. Specifically, subtle, moderate, and intense sweetness levels did not differ significantly from tastelessness, a finding corroborated by *post-hoc* analyses. This aligns with Eskine et al. (2011) study, which did not directly address the potential role of tastelessness. Consequently, we may hypothesize that, in daily life, simply drinking a glass of plain water might produce effects akin to those of sweet beverages.

However, this result necessitates further validation, especially given conflicting findings in a 2013 study (Hellmann et al., 2013), which may be due to differences in task types. Future research should employ neuroscience techniques, such as EEG or fNIRS, to record neural activity during online moral judgments, to further verify whether the tastelessness and sweetness groups activate the same brain regions or evoke comparable changes in EEG waveforms and potential distributions. Moreover, cultural factors could offer an alternative interpretation for the observed effects. In Chinese culture, the “tastelessness” of plain water might evoke aesthetic experiences among individuals. The tradition of using taste to metaphorize poetry and painting underscores this aesthetic narrative. In daily life, “tastelessness” is often associated with a calm, serene, and authentic lifestyle. Hence, under the Chinese cultural context, tastelessness may elicit a sense of calmness and aesthetic appreciation among participants.

Notably, this investigation provides the first evidence of a marked deviation in moral evaluations under suprathreshold sweetness conditions. As illustrated in Figures 2C,D, participants in the intense sweetness group exhibited significantly lower ratings for slight events compared to certain bitter taste subgroups. This paradoxical pattern suggests that excessive sweetness may induce aversive reactions akin to bitterness, potentially activating shared avoidance mechanisms (Chapman et al., 2009).

### 4.2 The varied effects of bitterness on judgments of different online events: a notable divergence in the intense bitterness group

In judging online events, significant differences were observed between the tastelessness group, the sweetness group, and the bitterness group. The data indicated that the impact of bitterness priming on the evaluation of online events was intricate, as different levels of bitterness influenced judgments of different types and severities of online events in varying ways.

For immoral online events, evaluations became increasingly stringent as the intensity of bitterness escalated from slight to moderate, with the moderate bitterness group exhibiting the strictest stance among all seven groups, as illustrated in Figure 2B. This result is consistent with Chapman et al. (2009), who demonstrated that bitterness activates neural circuits (e.g., the insula and amygdala) shared with moral disgust, thereby intensifying negative evaluations of immoral acts. However, under intense bitterness, a shift occurred, where participants showed greater leniency in their judgment of immoral online events. This reversal effect may stem from cultural differences: it found that bitterness differentially impacts decision-making speed across cultures (faster click intervals in UK samples vs. slower in Vietnamese samples) (Vi and Obrist, 2018), suggesting cultural context modulates bitterness-induced cognitive effects. Our findings further indicate that the Chinese philosophical metaphor of “enduring bitterness leads to sweetness” may trigger psychological compensation mechanisms under high-intensity bitterness, prompting individuals to adopt lenient judgments to alleviate physiological discomfort (Li et al., 2023).

Conversely, for online moral events, the trend reversed—participants became more lenient in their judgments as bitterness increased from slight to moderate, with the moderate bitterness group’s evaluations approaching those of the tastelessness and mild-to-moderate sweetness groups. Yet, under intense bitterness, judgments became much stricter. This aligns with neurophysiological explanations from an ERP study: bitterness first reduces visual attention to general social cues (diminished P200 amplitude) and subsequently specifically inhibits processing of non-rejection cues (reduced P300 amplitude) (Schienle et al., 2017). Such attentional resource allocation patterns may lead to excessive scrutiny of moral events—when bitterness intensity exceeds thresholds, individuals tend to overlook positive contextual cues and adopt stricter standards.

When assessing severe moral or immoral events, the intense bitterness group exhibited heightened tolerance toward immoral events while adopting stricter standards for moral events, compared to the other six groups. Specifically, the moderate bitterness group was the strictest in judging obviously severe immoral events, though its evaluations were comparable to the other groups when considering mildly severe events. This partially corroborates Sagioglou and Greitemeyer’s hostility enhancement model (Sagioglou and Greitemeyer, 2014). However, our study reveals that when bitterness intensity exceeds thresholds, hostility may be culturally reframed into moral standard adjustments rather than direct aggression. Notably, our study found that the intense bitterness group exhibited distinct differences from the other groups across all conditions, demonstrating greater leniency toward immoral events and stricter judgments of moral events, regardless of the level of severity. This pattern mirrored the intense sweetness group in mild events, although the intense sweetness group did not exhibit the same influence in severe events.

Our research revealed that taste priming had distinct effects on different types of online events, except for the intense bitterness group, where *post-hoc* analyses indicated some differences. This complexity may also be tied to Chinese cultural contexts, where bitterness holds unique interpretations. In Chinese culture, “bitterness” extends beyond a simple taste descriptor or a reflection of life conditions; it embodies philosophical aspects of life. While bitter-tasting drinks may induce a sense of disgust, this feeling does not necessarily translate into moral disgust. Instead, the negative self-perception triggered by bitterness could lead individuals to adopt more lenient moral standards as a means of self-repair (Li et al., 2023). Future research should continue to explore the impact of and bitterness on aesthetic judgment.

### 4.3 Differential effects of taste types on the evaluation of events with different levels of severity

Our research identified differential effects of taste types on the evaluation of online events with varying levels of severity (mild and obvious). The influence of taste on the evaluation of mildly severe events was marginally significant, while its effect on obviously severe events was more pronounced. For mildly severe events, both intense sweetness and intense bitterness exhibited differential impacts compared to other groups, although not all differences were statistically significant.

However, in obviously severe events, the differences observed in the intense sweetness group diminished, while the intense bitterness group continued to show significant differences from each of the other groups. The findings suggest that participants’ evaluations of obviously severe events were higher than those of mildly severe events, with the level of severity exerting a more substantial impact on the judgment of immoral events than moral ones. Therefore, in the governance of cyberspace, recognizing the severity level of online events is crucial. Different strategies must be employed for different levels of event severity to ensure that desired governance outcomes are achieved.

### 4.4 A new perspective on cyberspace governance: the impact of taste types on online event judgment

The conclusions of our study provide practical insights into how the selection of various taste intensities in daily life can influence our perceptions of network events and the real world. Furthermore, our research offers detailed descriptions regarding the composition, dosage, and priming procedures of bitterness and sweetness, which will facilitate the comparability and reproducibility of future studies.

Regarding the regulation of cyberspace, while legal measures are undoubtedly important, this study introduces a new perspective on the matter. Faced with various online incidents, if internet users were to sip a beverage of different flavors, it could shape diverse moral evaluations, potentially leading to a reduction in behaviors that violate online ethics and challenge legal boundaries.

When citizens engage in cyberspace discussions, whether on mild or severe moral events, such as evaluating “bystander apathy” (Hortensius and de Gelder, 2018), navigating the dichotomy between “procedural justice and substantive justice,” or discussing education - related issues (Ma et al., 2025a; Ma et al., 2025b), or evaluating the severity of punishment for “buyers involved in human trafficking” (Niemi and Aaltonen, 2017)—consuming a cup of intensely bitter beverage, like black coffee, could evoke empathy, encourage perspective-taking under adverse conditions, and lead to more lenient judgments of immoral events. Conversely, a glass of plain water or a lightly flavored drink might prompt us to appraise positive events more favorably, thereby fostering prosocial behaviors.

### 4.5 Limitations and implications

*Limitations*: this research has several limitations that warrant consideration. First, the experimental sample was restricted to Chinese university students, whose homogeneous age distribution (approximately 20 years old) and sociocultural backgrounds may constrain the generalizability of findings to broader populations. Second, while standardized gustatory stimuli (sucrose and bitter melon powder) were employed, the bitter melon powder may contain non-bitter compounds (e.g., alkaloids), leaving open the possibility of confounding cognitive effects independent of bitterness perception. Third, the laboratory setting and simplified moral judgment tasks—using predefined text-image pairs—may lack ecological validity, as they fail to capture the dynamic, interactive nature of real-world online events. Additionally, individual taste preferences and cultural-specific interpretations of bitterness (e.g., resilience metaphors in Chinese philosophy) were not systematically controlled, potentially influencing outcome variability. Finally, the transient nature of gustatory priming was not assessed, leaving the durability of taste-induced effects on online moral judgment unexplored.

*Implications*: this research proposes an innovative sensory intervention framework for cyberspace governance. By modulating users’ gustatory experiences (e.g., intense bitterness evoking empathetic tolerance, tastelessness promoting prosocial evaluations), it supplements traditional legal approaches to indirectly guide online moral decision-making. Specifically, platforms can implement taste-adaptive strategies—recommending sweet beverages in contentious discussion zones to reduce aggressive rhetoric, or leveraging bitterness to foster perspective-taking in policy debates—thereby cultivating rational discourse and mitigating ethical violations. These findings directly inform the development of culturally sensitive interface designs (e.g., taste-triggered alert systems) and digital literacy tools (e.g., VR-based taste-moral decision simulations), offering actionable pathways to enhance online civility and self-regulation.

## Data Availability

The datasets presented in this study can be found in online repositories. The names of the repository/repositories and accession number(s) can be found in the article/supplementary material.
